# Digital Alcohol Interventions Could Be Part of the Societal Response to Harmful Consumption, but We Know Little About Their Long-Term Costs and Health Outcomes

**DOI:** 10.2196/44574

**Published:** 2024-03-27

**Authors:** Katarina Ulfsdotter Gunnarsson, Martin Henriksson, Marcus Bendtsen

**Affiliations:** 1 Department of Health, Medicine, and Caring Sciences Linköping University Linköping Sweden

**Keywords:** alcohol, health economics, telemedicine, psychological harm, eHealth, digital intervention, decision-making

## Abstract

Alcohol consumption causes both physical and psychological harm and is a leading risk factor for noncommunicable diseases. Digital alcohol interventions have been found to support those looking for help by giving them tools for change. However, whether digital interventions can help tackle the long-term societal consequences of harmful alcohol consumption in a cost-effective manner has not been adequately evaluated. In this Viewpoint, we propose that studies of digital alcohol interventions rarely evaluate the consequences of wider dissemination of the intervention under study, and that when they do, they do not take advantage of modeling techniques that allow for appropriately studying consequences over a longer time horizon than the study period when the intervention is tested. We argue that to help decision-makers to prioritize resources for research and dissemination, it is important to model long-term costs and health outcomes. Further, this type of modeling gives important insights into the context in which interventions are studied and highlights where more research is required and where sufficient evidence is available. The viewpoint therefore invites the researcher not only to reflect on which interventions to study but also how to evaluate their long-term consequences.

## Beyond the Buzz: Unraveling the Nonexistent Long-Term Outcomes

Consumption of alcohol is in many societies the norm [1]. In Sweden, for instance, alcohol is consumed regularly by 4 of 5 adults, and approximately 1 of 3 adults are classified as harmful drinkers [2,3]. While evidence suggests that there is no safe limit on alcohol consumption [4], harmful drinking represents a marked increased risk of negative health and social consequences [1,4,5]. In 2016, alcohol was estimated to have contributed to approximately 6.2% of disability-adjusted life years (DALYs) among women and 5.1% of DALYs among men in Sweden, and was attributed to 4.5% and 5.7% of deaths (women and men, respectively) [1]. A health economic report showed that in 2017, alcohol consumption cost Swedish society €10 billion [6]. Sweden is not unique in this situation, as many societies worldwide face significant health and economic burdens due to the high prevalence of harmful alcohol consumption.

Reducing the harmful use of alcohol is on the World Health Organization’s list of “best buys” for tackling noncommunicable diseases [7]. Recommended actions include excise taxes on alcoholic beverages and restrictions on the retail availability of alcohol. Provisioning of psychosocial interventions for persons with harmful alcohol use is also included on the list of recommended actions, and has been operationalized by, for instance, delivering face-to-face interventions in primary health care [8]. With the ubiquity of internet connectivity in high-income countries, and increasingly in low- and middle-income countries, there is interest to provide *digital* psychosocial interventions to those who may benefit. Digital interventions—that is, interventions that deliver supportive content through for instance websites, mobile phone apps, text messages, or email—can support those looking for help by giving them tools to increase their motivation, form intentions for change, and give them tools to help support change [9,10]. Digital interventions can scale to large populations and can be designed to be anonymous, which can reduce the stigma of looking for help in face-to-face settings [11-13]. Studies have found that digital alcohol interventions may have positive effects on behavior in a range of different populations [14-17], and evidence from meta-analyses confirms these findings [18-20]. However, whether or not they can help tackle the long-term consequences of harmful alcohol consumption, for instance by reducing the incidence of noncommunicable diseases, and whether or not they can do so in a cost-effective manner, is still uncertain. In contrast, modeling studies have demonstrated that face-to-face interventions hold promise in mitigating health consequences while being cost-effective over the long term [21]. Thus, limiting our assessment only to the short-term effects of digital interventions means that we cannot compare the societal impacts between modalities.

We propose that studies of digital alcohol interventions rarely evaluate the consequences of wider dissemination of the intervention under study, and that when they do, they do not take advantage of modeling techniques that allow for appropriately studying consequences over a longer time horizon than the study’s period when the intervention is tested. We support our viewpoint with a pragmatic review of the literature (Multimedia Appendix 1 [22-27]) and make suggestions for how future studies may evaluate the impact of digital alcohol interventions at the societal level—which includes workplace and productivity, social services and nonstatutory care, and the criminal justice sector—and not only consequences related to the health care sector such as costs for treatment. We also invite researchers to reflect on their decision-making when it comes to deciding which intervention research projects should be prioritized; in particular, if formal evaluations of the long-term consequences of dissemination are considered, or even required, in the current decision-making process.

## Our View

Health economic evaluations of digital alcohol interventions are scarce. When done, the consequences of interventions are evaluated over a short period, the period over which a randomized controlled trial (RCT) is run, ranging from 4 to 12 months. While the reports identified in our pragmatic review (Multimedia Appendix 1 [22-27]) all concluded that the interventions were cost-effective, they based these findings on short-term follow-up data collected during the trial period—it stands to reason that while there certainly are acute consequences from alcohol consumption, the long-term consequences are substantial and cannot be captured when evaluating such short time horizons [28]. Thus, current evaluations cannot readily answer the question being asked: What are the consequences of disseminating digital alcohol interventions into society? Considering the growth in interest and resources put into studying digital alcohol interventions, it is unfortunate that the literature cannot provide evidence of the long-term consequences of this investment.

The literature can however provide examples of long-term health economic evaluations outside the scope of digital interventions. Studies of the long-term consequences of nondigital alcohol interventions have been conducted, including the development and use of the Sheffield Alcohol Policy Model [29], which evaluates public health strategies for alcohol harm reduction. In addition, a review of nondigital brief alcohol interventions found that 14 out of the 23 included studies used modeling techniques that allowed for estimating consequences over a lifetime horizon. The majority of the 14 studies discovered that they either saved costs while improving health or incurred minimal costs compared to the health benefits [21]. Thus, there exist examples in the literature for evaluating alcohol interventions, which can inspire and be partially adopted by the digital intervention research field. Other literature can be followed to model long-term health economic outcomes, which guides the construction, analysis, and implementation of health economic models [30,31]. In addition, if it is important to model interactions among individuals, their propensity to use support, and relationships between multiple physical and mental health conditions, then agent-based models can be used to capture these complexities and forecast the long-term effects of interventions [32,33].

## Importance of Health Economic Research

The importance of health economic evaluations in general, and modeling studies in particular, should not be understated. They provide input to decision-makers faced with difficult prioritization tasks, that is, if resources should be invested in disseminating a specific intervention. In many jurisdictions with a publicly funded health care system, they have long been an integral part of health policy [34] with a broad acceptance that modeling methods are required [35], not least in the evaluation of cancer drugs where intermediate outcomes (eg, progression-free survival) are routinely extrapolated to outcomes such as mortality [36]. It seems prudent that public health care efforts also should provide similar input of long-term outcomes since they often fall under the jurisdiction of public health care systems, however, this is not the case today. Their role can further be extended to also guide the prioritization of research resources. In developing an intervention, one may consider if a hypothetical intervention with a potential effect size even is worth developing and evaluating in an RCT. If such a hypothetical intervention cannot be shown to be cost-effective, then there is no reason to invest resources in its development and evaluation. Researchers are routinely asked by review panels to provide sample size estimates, which are based on anticipated effect sizes. These anticipated effect sizes commonly fall into 2 categories [37-39]: so-called clinical significance, which presents effect sizes large enough that the anticipated sample size is achievable, or minimum relevant effect size, which ensures that even small effect estimates are identified as statistically significant. Interestingly, these anticipated effect sizes are rarely (if ever) put to the test in a health economic evaluation where their long-term costs and health implications can be estimated; thus, a rationale for a study based on a formal evaluation of the long-term consequences of dissemination of the proposed intervention is not produced. We argue that health economic evaluations before RCTs can help reduce research waste and allow both researchers and other decision-makers to prioritize resources more effectively and, indeed, even identify cost-effective research designs [40].

It should be acknowledged that conducting health economic evaluations is not without barriers. It requires a certain technical skill set by the researcher conducting the evaluation, usually involving statistical software to create models that can extrapolate over time. It also requires epidemiological expertise to decide on input variables, as well as knowledge of the costs and consequences from both the health care and societal perspectives concerning treatment and disease. These are formidable challenges; however, approaching these challenges confers additional benefits. First, it puts into context the intervention being studied, allowing researchers to carefully think about the degree to which interventions will be adopted, their anticipated short- and long-term costs and effects, and how the novel intervention relates to existing interventions. Second, it makes it very clear where evidence is lacking from both an efficacy, epidemiologic, and economic perspective. For instance, when faced with having to decide on the risk of disease given a range of contextual variables, the process of conducting a health economic evaluation highlights where epidemiological studies are required, but also where the evidence is sufficient. Thus, by conducting a health economic evaluation, research prioritizations can become clear beyond the primary objective of the study. These prioritizations can be further refined by conducting sensitivity analyses to show which input variables affect the outcome of the evaluation the most, refining the agenda for which phenomena require further study. Third, by taking our proposed route, one that has been the gold standard for a long time in clinical medicine, decision-makers will be provided with the relevant evidence when prioritizing scarce resources that can be used for the provision of interventions or for further research. Basing evaluations solely on data from 1 RCT will yield a very precise estimate of an irrelevant cost-effectiveness estimate; thus, our advice is to follow the old advice: It is better to be roughly right than precisely wrong.

To conclude, health economic evaluations of digital alcohol interventions lack modeling of long-term societal consequences, leaving a knowledge gap concerning the degree to which they may address the burgeoning societal burdens caused by alcohol consumption. We invite researchers to reflect on their decision-making process when it comes to deciding which studies should be prioritized.

## Appendix

Multimedia Appendix 1 A pragmatic review.

## Abbreviations

DALY: disability-adjusted life year
RCT: randomized controlled trial
