# Supplementary material for: Digital Alcohol Interventions Could Be Part of the Societal Response to Harmful Consumption, but We Know Little About Their Long-Term Costs and Health Outcomes
Source: J Med Internet Res. 2024 Mar 27;26:e44574. doi: 10.2196/44574 (PMC11007605; doi:10.2196/44574)
Supplement: Multimedia Appendix 1 [file jmir_v26i1e44574_app1.docx]

# appendix A - A pragmatic review

To support our proposition that studies of digital alcohol interventions rarely evaluate the consequences of a wider dissemination of the intervention under study, we conducted a systematic search in PubMed for reports of health economic evaluations of digital alcohol interventions was conducted on 2022-02-22 (please see section Search Query for the keywords used). On 2023-03-03, an additional search in Clinicaltrials.gov was conducted using search terms "digital alcohol interventions and cost-effectiveness" and "alcohol and health economic evaluation" but no relevant studies appeared. Studies were excluded if they did not study a digital intervention, studied other behaviors than alcohol or included alcohol with other substances, were a pilot study, or did not include any health economic evaluation. There were no exclusion criteria based on the characteristics of study participants or the setting in which the study was conducted, but articles had to be in the English language.

Authors KUG and MB independently reviewed titles and abstracts of all reports. Studies which were kept after the initial pruning were combined and read in full by both KUG and MB. For reports where KUG and MB were not in agreement regarding inclusion after reading the full text, the third author MH acted as arbiter. Information on the following data items were extracted by author KUG:

- Description of the digital intervention
- Comparator
- Health economic evaluation method (e.g., randomized control trials (RCT), decision model, etc.)
- Effect measurements (e.g., quality-adjusted life years (QALYs), alcohol consumption, etc.)
- Time frame of analysis
- Perspective on cost and benefits (e.g., health care, wider societal, etc.)

The extracted information and the full text of the published reports were used to produce a narrative synthesis.

## Search QUERY

The search query used to identify reports in PubMed is presented below:

("alcohol"[Title/Abstract] OR "beverages"[Title/Abstract] OR "beverages"[MeSH Terms] OR "beer"[Title/Abstract] OR "beer"[MeSH Terms] OR "wine"[Title/Abstract] OR "wine"[MeSH Terms] OR "cider"[Title/Abstract] OR "alcopop"[Title/Abstract] OR "spirit"[Title/Abstract] OR "spirits"[Title/Abstract] OR "standard glass"[Title/Abstract] OR "binge"[Title/Abstract] OR "drink"[Title/Abstract] OR "drinking"[Title/Abstract] OR "drinking"[MeSH Terms] OR "drinkers"[Title/Abstract] OR "intoxication"[Title/Abstract] OR "inebriated"[Title/Abstract] OR "drunk"[Title/Abstract])

AND

("intervention"[Title/Abstract] OR "program"[Title/Abstract] OR "support"[Title/Abstract] OR "tool"[Title/Abstract] OR "promotion"[Title/Abstract] OR "education"[Title/Abstract] OR "education"[MeSH Terms] OR "advice"[Title/Abstract] OR "improve"[Title/Abstract] OR "prevention"[Title/Abstract] OR "treatment"[Title/Abstract] OR "manage"[Title/Abstract] OR "management"[Title/Abstract] OR "managing"[Title/Abstract] OR "alter"[Title/Abstract] OR "altering"[Title/Abstract] OR "modification"[Title/Abstract] OR "modifying"[Title/Abstract] OR "change"[Title/Abstract] OR "changing"[Title/Abstract] OR "reduction"[Title/Abstract] OR "cessation"[Title/Abstract])

AND

("web"[Title/Abstract] OR "web-based"[Title/Abstract] OR "website"[Title/Abstract] OR "homepage"[Title/Abstract] OR "home page"[Title/Abstract] OR "online"[Title/Abstract] OR "internet"[Title/Abstract] OR "internet"[MeSH Terms] OR "computer"[Title/Abstract] OR "computer-tailored"[Title/Abstract] OR "computer-based"[Title/Abstract] OR "email"[Title/Abstract] OR "e-mail"[Title/Abstract] OR "electronic mail"[Title/Abstract] OR "electronic mail"[MeSH Terms] OR "digital"[Title/Abstract] OR "telemedicine"[Title/Abstract] OR "telemedicine"[MeSH Terms] OR "mhealth"[Title/Abstract] OR "m-health"[Title/Abstract] OR "mobile health"[Title/Abstract] OR "ehealth"[Title/Abstract] OR "e-health"[Title/Abstract] OR "electronic health"[Title/Abstract] OR "mobile"[Title/Abstract] OR "mobile phone"[Title/Abstract] OR "mobile application"[Title/Abstract] OR "mobile applications"[Title/Abstract] OR "mobile applications"[MeSH Terms] OR "mobile phone-based interventions"[Title/Abstract] OR "mobile messages"[Title/Abstract] OR "mobile messaging"[Title/Abstract] OR "smartphone"[Title/Abstract] OR "smartphone"[MeSH Terms] OR "app"[Title/Abstract] OR "apps"[Title/Abstract] OR "application"[Title/Abstract] OR "ecological momentary intervention"[Title/Abstract] OR "EMI"[Title/Abstract] OR "telephone"[Title/Abstract] OR "telephone"[MeSH Terms] OR "cell phone"[Title/Abstract] OR "cell phone"[MeSH Terms] OR "text message"[Title/Abstract] OR "text messages"[Title/Abstract] OR "text messaging"[Title/Abstract] OR "text messaging"[MeSH Terms] OR "texting"[Title/Abstract] OR "short message"[Title/Abstract] OR "short messages"[Title/Abstract] OR "short message service"[Title/Abstract] OR "SMS"[Title/Abstract])

AND

("Costs and cost analysis"[MeSH Terms] OR "markov chains"[MeSH Terms] OR ("cost"[Title/Abstract] AND "benefit"[Title/Abstract]) OR ("cost"[Title/Abstract] AND "effective*"[Title/Abstract]) OR ("economic"[Title/Abstract] AND "evaluation"[Title/Abstract]) OR ("cost"[Title/Abstract] AND "utility"[Title/Abstract]) OR "markov"[Title/Abstract])

# Results

PubMed delivered a total of 925 items, of which 19 studies were eligible for full-text review (see Figure 1). Authors KUG and MB independently pruned the full search based on the title and the abstract and identified 19 reports in total. The reports that did not make it to full text review were missing a health economic evaluation or were not related to digital interventions. KUG and MB assessed the 19 articles together in full text in accordance with the eligibility criteria and excluded 13 reports. There were no disagreements which required arbitration from the third author. There were 6 reports that included a health economic evaluation of a digital alcohol intervention. Table 1 presents an extraction of the data and shows that the 6 identified reports included a health economic evaluation based solely on an RCT; either as a re-analysis of a previously reported trial or as part of the primary report of the trial. Note that one of the reports was a protocol with the intention to evaluate an intervention in an RCT, including a cost-effectiveness analysis.


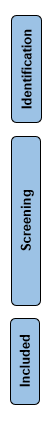


Records identified from:

PubMed (n = 925)

Clinical trials (n = 0)

Full-text articles excluded (n = 13)

- No digital tool/too small component was digital (n = 4)

- Not only alcohol (n = 7)

- No health economic evaluation (n = 1)

- Pilot study (n = 1)

Abstracts retained for full text review (n = 19)

Articles included in review
(n = 6)

**Figure 1. PRISMA flow diagram**

Generic health outcomes, QALYs or DALYs, were used in four studies and self-reported alcohol measurements, such as AUDIT-C and weekly alcohol consumption were used in three. One study reported both effect measures. The three studies that used QALY collected quality-of-life weight using the instrument EQ-5D-5L.

All studies covered the study period of the RCT, ranging from four months to 12 months. No studies modelled behavior, disease, and costs, which would have allowed for extrapolation beyond the study period. Most commonly a societal perspective was taken, with two reports taking a health care perspective. The societal perspectives taken included costs for the intervention, health care, productivity loss, costs for education, labour, social security, household and leisure, and criminal justice systems.

Table 1 – Extracted data from reports of health economic evaluations of digital alcohol interventions

|  | **Intervention** | **Comparison** | **Setting/Population** | **Analytic method** | **Effect measurements** | **Time frame** | **Perspective on cost and benefits** |
| --- | --- | --- | --- | --- | --- | --- | --- |
| Drost et al. 2016 [22] | Web-based computer-tailored intervention: game with tailored feedback on alcohol awareness | Care as usual (waiting list) | Dutch adolescents attending school (15-19 years) | RCT | Weekly alcohol use and the number of binge drinking occasions | 4 m | Societal + health care |
|  |  |  |  |  |  |  |  |
| Deluca et al. 2021 [23] | Face-to-face personalised feedback and brief advice + electronic brief intervention | Screening only | UK emergency departments  (14-17 years) | RCT | AUDIT-C + QALY (EQ-5D-5L) | 12 m | Societal |
|  |  |  |  |  |  |  |  |
| Blankers et al. 2012 [24] | Internet-based therapy: text-based chat-therapy sessions + home assignment | Internet-based self-help | Dutch adults  (18-65 years) | RCT | QALY (EQ-5D-5L) | 6 m | Societal |
|  |  |  |  |  |  |  |  |
| Mujcic et al. 2022 [25] | A digital alcohol moderation intervention: website with consumption plan, exercises, self-monitoring + per support platform | Health information | Dutch adults  (10-year cancer survivors) | RCT | Self-reported number of standard drinks, AUDIT, QALY (EQ-5D-5L) | 12 m | Societal |
|  |  |  |  |  |  |  |  |
| Boß et al. 2015 [26] | Web-based self-help for problematic drinking, adherence-focused guided self-help | Waiting list | German adults  (18+ years) | RCT* | Self-reported alcohol consumption | 6 m | Employers and society |
|  |  |  |  |  |  |  |  |
| Smit et al. 2012  [27] | Face-to-face interventions and eHealth interventions | Care as usual | Dutch adults  (18-69 years) | RCT | DALY | 12 m | Health care |
| * Study protocol | | | | | | | |
